# Drug target Mendelian randomisation: are we really instrumenting drug use? Reply to Anderson EL, Williams DM [letter]

**DOI:** 10.1007/s00125-023-05892-w

**Published:** 2023-03-10

**Authors:** Jie Zheng, Tom R. Gaunt, Min Xu, George Davey Smith, Yufang Bi

**Affiliations:** 1 Department of Endocrine and Metabolic Diseases, Shanghai Institute of Endocrine and Metabolic Diseases, Ruijin Hospital, Shanghai Jiao Tong University School of Medicine, Shanghai, China; 2 Shanghai National Clinical Research Center for Metabolic Diseases, Key Laboratory for Endocrine and Metabolic Diseases of the National Health Commission of the PR China, Shanghai Key Laboratory for Endocrine Tumor, Shanghai Digital Medicine Innovation Center, Ruijin Hospital, Shanghai Jiao Tong University School of Medicine, Shanghai, China; 3 MRC Integrative Epidemiology Unit (IEU), Bristol Medical School, University of Bristol, Bristol, UK

**Keywords:** Dementia, Drug target, Mendelian randomisation, Metformin

*To the Editor*: We appreciate the opportunity to respond to the letter by Anderson and Williams [1] on our recent article on evaluating the effects of metformin drug targets on Alzheimer’s disease [2]. As experts in the dementia research field, Anderson and Williams share our opinion that drug target Mendelian randomisation (MR) is a promising approach to repurposing drugs for prevention in Alzheimer’s disease [3]. This is particularly important as there are currently no promising drug interventions for Alzheimer’s disease prevention, and clinical trials of Alzheimer’s disease prevention need to run for many years to be informative [1].

We thank Anderson and Williams [1] for pointing out the issue with the term ‘genetically proxied metformin use’. In our paper [2], we used genetics to proxy the genetic effect of each of five identified metformin drug targets on lowering HbA_1c_ levels. We then used a fixed-/random-effect meta-analysis approach to summarise the effect estimates. Therefore, a more accurate interpretation of the exposure in this study is a genetically proxied inverse variance weighted HbA_1c_-lowering effect of the five metformin targets, rather than actual metformin use. We hope this will help clarify this point.

In our study, we had a similar vision to Anderson and Williams [1] about the value of considering individual metformin drug targets. As we pointed out in our paper, the biology of metformin is still only partially understood and it is possible that some novel or less studied targets were missed. For example, a recent study suggested *PEN2* as a direct molecular target of metformin when the dose is low [4], which could be investigated in future MR studies. However, our study provides a starting point for exploring this complex scientific question; we hope it will attract further attention from experts in the field and provide some biological/methodological clues for future studies of metformin targets.

In response to the issue raised by Anderson and Williams [1] of averaging across targets, our study had two major aims: (1) to estimate the individual effect of each metformin target and compare their effects on Alzheimer’s disease; (2) to explore the combined effect of all tested metformin targets on Alzheimer’s disease. The first aim was achieved in our study, in which we showed that all five targets have a predicted effect on Alzheimer’s disease in a protective direction. This result suggests a combined effect of independent targets, but does not prove that this occurs via glucose lowering. We also agree with Anderson and Williams [1] that accurate quantification of the combined effect is challenging and that our averaging method does not consider differential effects of metformin on those targets. We also considered other issues. For example, two of the targets we studied, AMP-activated protein kinase (AMPK) and mitochondrial complex I, may share a similar biological pathway, which means they may interact with each other. In addition, it is possible that the overall effect is double counted in single target MR if multiple targets (e.g. AMPK and presenilin enhancer 2 [PEN2] [4]) function at two levels in the same biological pathway. However, currently available data do not allow us to answer these questions. Therefore, in future drug target MR analysis of multi-targets drugs such as metformin, the true biology (e.g. function of the targets, the interaction between targets and upstream/downstream relationships of targets in the same biological pathway) needs to be considered very carefully. Novel method development based on multivariable MR and factorial MR and new data on the differential effects of drugs on targets are also needed to truly proxy the overall effects of complex drugs such as metformin.

The direct comparison of MR, clinical trial and observational data is challenging and we acknowledge in our paper and electronic supplementary material (ESM) some of the limitations that Anderson and Williams [1] highlight. However, it is still useful to consider whether these methods show the same direct of effect. Combining our results with existing epidemiology evidence that metformin use may reduce the incidence of dementia [5, 6], we consider that the ‘worst-case scenario’ of a harmful effect of metformin use or metformin targets on dementia/Alzheimer’s disease, as raised in the letter by Anderson and Williams, is unlikely.

In addition, we would like to clarify that drug target MR may show different effects from MR of the proxied biomarker. For example, a recent drug target MR analysis of the association between *HMGCR* inhibition (proxied by LDL-cholesterol levels) and ovarian cancer identified a strong protective effect. However, there is little evidence to support an effect of LDL-cholesterol on ovarian cancer [7]. Therefore, it is possible that glycaemic control may not have a direct effect on dementia prevention, as our sensitivity analysis of the MR effects of HbA_1c_ on Alzheimer’s disease suggests (see ESM Table 10C in Zheng et al [2]). This motivated us to conduct the expression quantitative trait locus (eQTL) MR analysis on Alzheimer’s disease, in which we identified some potential novel drug targets, for example *NDUFA2*, for Alzheimer’s disease prevention.

We agree with Anderson and Williams [1] that the use of ‘Alzheimer’s disease-by-proxy’ phenotypes may increase the influence of misclassification bias. The genetic data we used for Alzheimer’s disease combined clinically defined Alzheimer’s disease cases with Alzheimer’s disease-by-proxy cases to increase statistical power, but at the cost of potential misclassification. It will be important to repeat our analysis as more powerful and specific Alzheimer’s disease genome-wide association study (GWAS) datasets become available.

Finally, we agree with Anderson and William’s point that survival bias may have affected our results, but to a lesser extent than in MR of other (non-molecular) phenotypes [1].

We would like to thank Anderson and Williams for a detailed evaluation of our paper, highlighting some of the key challenges for research in this area. We hope this exchange will assist readers in interpreting our results and inform future genetic studies of metformin targets. We also look forward to concrete suggestions on how to improve the field being advanced.

## Abbreviations

AMPK: AMP-activated protein kinase
MR: Mendelian randomisation
